# Liver transplantation after DRESS syndrome: A case report and review of the literature

**DOI:** 10.1002/ccr3.3334

**Published:** 2020-09-15

**Authors:** Igor Lepski Calil, Francisco Tustumi, Rafael Soares Nunes Pinheiro, Ryan Yukimatsu Tanigawa, Ruy Jorge Cruz Junior, Jorge Henrique Bento de Sousa, Rafael Antônio Arruda Pecora, Luiz Augusto Carneiro D'Albuquerque

**Affiliations:** ^1^ Hospital Israelita Albert Einstein Sao Paulo Brazil; ^2^ Universidade de São Paulo Sao Paulo Brazil; ^3^ Centro Universitário Lusíada Santos Brazil

**Keywords:** case reports, drug hypersensitivity syndrome, liver failure, sulfasalazine

## Abstract

This study reports a patient with DRESS syndrome, associated with liver failure, treated with orthotopic liver transplantation.

## Graphical abstract

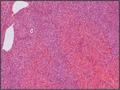

## 1 INTRODUCTION

Drug reaction with eosinophilia and systemic symptoms is an unusual drug reaction–related condition. A case report of sulfasalazine‐induced liver failure is described. The patient required liver transplantation. Liver transplantation is an option when the DRESS syndrome is associated with acute liver failure, but the prognosis remains poor.

Drug reaction with eosinophilia and systemic symptoms (DRESS syndrome) is a rare drug reaction–related condition.^1^ Several drugs have been linked to DRESS. It is a severe idiosyncratic drug reaction characterized by erythematous or papulopustular skin eruption associated with lymphadenopathy, fever, and visceral involvement (hepatitis, nephritis pneumonitis, pericarditis, myocarditis, and colitis).^2^, ^3^, ^4^, ^5^, ^6^ Leukocytosis, eosinophilia (90%), or mononucleosis (40%) may also be identified.^6^ Severe acute hepatitis due to sulfasalazine or trimethoprim‐sulfamethoxazole is described in the literature, but the occurrence of DRESS syndrome causing liver failure is rare.

In this study, we report a patient with acute liver failure due to sulfasalazine‐induced DRESS, treated with a liver transplant.

## 2 CASE REPORT

An 18‐year‐old male patient was treated with sulfasalazine for toxoplasma retinochoroiditis for one month. The patient had no history of allergies or drug intolerance. He reported to a local hospital with fever, vomiting, cervical and inguinal nodules, abdominal pain, and generalized body macular rash. The patient was transferred to our transplant center after the onset of jaundice and encephalopathy. He was admitted to the intensive care unit with facial edema, generalized scaling exanthema, and acute hepatitis. Serological tests for viral hepatitis and all autoimmune antibodies were negative. Laboratory tests showed a total eosinophil count of 3220/mm^3^ (normal < 500 mm^3^), high level of transaminases (aspartate aminotransferase = 1303 IU/L; alanine aminotransferase = 1768 IU/L, lactate dehydrogenase level of 2274 IU/L (normal, 240‐480 IU/L), total bilirubin level of 18.47 mg/dL, direct bilirubin level of 14.81 mg/dL, prothrombin time (PT), international normalized ratio (INR) of 5.18, and factor V 17% (normal, 50%‐150%). The MELD score was 43.

An abdominal ultrasound examination identified no chronic liver disease. The RegiSCAR (Registry of Severe Cutaneous Adverse Reaction)^7^ system scored 5 points, confirming the DRESS syndrome diagnosis. Skin biopsy observed interface and spongiotic dermatitis, consistent with drug eruption.

Following the Clichy criteria,^8^ the patient was worked up for urgent orthotopic liver transplantation (OLT), which was performed 24 hours after admission. At the time, he was under corticosteroids and clinical support, including mechanic ventilation due to progressive encephalopathy and dialysis due to lactic acidosis.

The orthotopic liver transplantation was uneventful. While liver function improved in the postoperative period, the patient developed sepsis requiring high doses of vasopressors. Broad‐spectrum antibiotics were introduced (third‐generation cephalosporin associated with ampicillin and then switched to carbapenem), but the patient remained hemodynamically unstable. The laboratory test showed progressively increasing acidosis and lactate levels, aspartate aminotransferase = 230 IU/L, alanine aminotransferase = 110 IU/L, total bilirubin level of 2 mg/dL, and INR of 1.5. The patient died on the seventh postoperative day. Blood cultures showed growth of *Klebsiella pneumoniae* resistant to carbapenems.

### 2.1 Liver and skin histology

**Figure 1:**
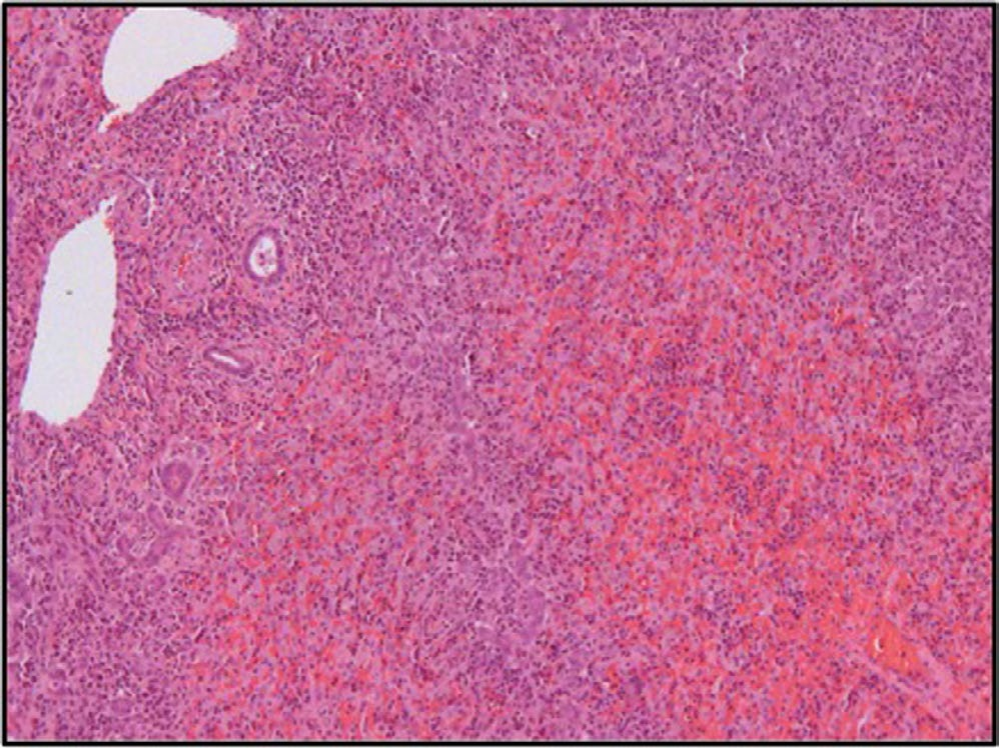
Liver histology. H&E stain. Massive eosinophilic infiltrate with extensive necrosis of the liver compatible with fulminant hepatitis

**Figure 2:**
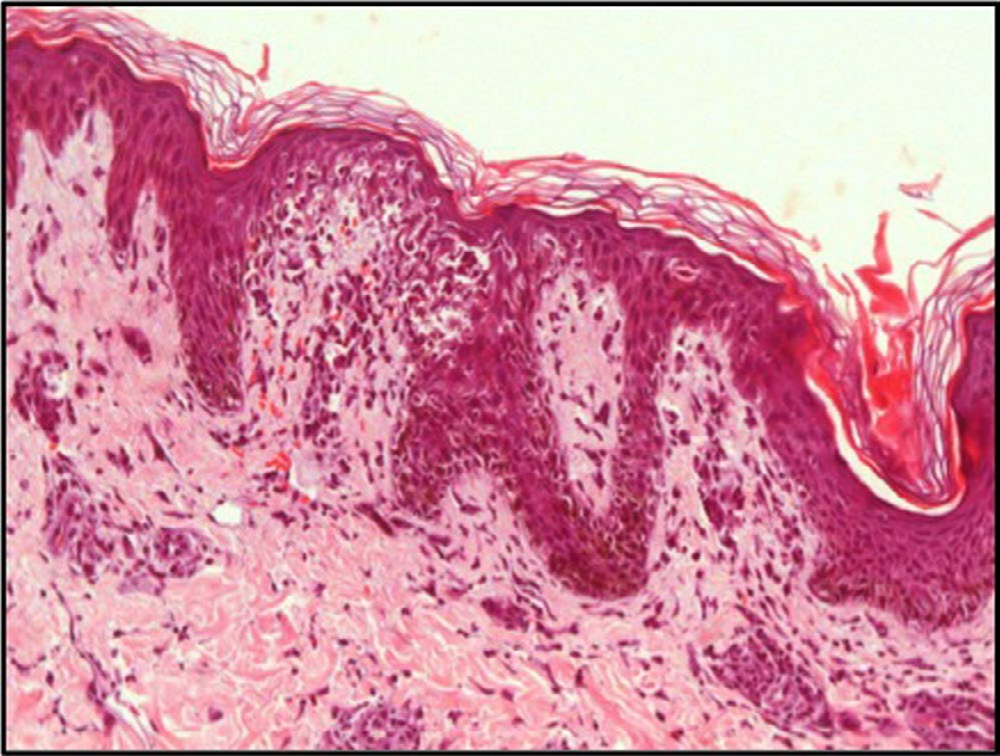
Skin histology. H&E stain. Massive eosinophilic infiltrate compatible with DRESS syndrome

The histological analysis demonstrated massive eosinophilic infiltrates compatible with the diagnosis of DRESS syndrome, and the liver explant showed massive necrosis associated with eosinophilic infiltrate (Figures 1 and 2).

## 3 DISCUSSION

This report describes an adult patient with DRESS syndrome and liver failure treated with OLT. Liver failure in the setting of DRESS syndrome is quite rare. A few case reports recorded in the literature showed DRESS syndrome and significant hepatic injury (Table 1). In these studies, most of the patients were treated with corticosteroids.^9^, ^10^, ^11^, ^12^, ^13^, ^14^, ^15^, ^16^, ^17^, ^18^, ^19^, ^20^, ^21^, ^22^, ^23^, ^24^, ^25^, ^26^, ^27^, ^28^, ^29^, ^30^ Among patients needing liver transplantation, there was approximately 50% perioperative mortality.

**Table 1:** Reported cases who had diagnosis of DRESS syndrome caused by associated sulfasalazine and trimethoprim‐sulfamethoxazole

Case report	Patient	Drug	Treatment	Follow‐up
Brooks H et al^9^	53‐year‐old man	Sulfasalazine	Corticosteroids	Alive
Queyrel V et al^10^	15‐year‐old girl	Sulfasalazine	Corticosteroids	Alive
Mainra RR et al^11^	24‐year‐old woman	Trimethoprim‐sulfamethoxazole	Corticosteroids	Alive
Descloux E et al^12^	45‐year‐old woman	Sulfasalazine	Corticosteroids	Alive
Michel F et al^13^	63‐year‐old woman	sulfasalazine	Corticosteroids	Alive
Teo L et al^14^	49‐year‐old woman	Sulfasalazine	Corticosteroids	Alive
Bejia I et al^15^	46‐year‐old woman	Sulfasalazine	Corticosteroids	Alive
de Aquino RT et al^16^	47‐year‐old woman	Sulfasalazine	Corticosteroids	Alive
Augusto JF et al^17^	77‐year‐old woman	Sulfasalazine	Corticosteroids	Alive
Yeşilova Z et al^18^	38‐year‐old man	Sulfasalazine	Corticosteroids	Alive
Rosenbaum J et al^19^	11‐year‐old girl	Sulfasalazine	Corticosteroids	Alive
van der Mark SC et al^20^	24‐year‐old woman	Sulfasalazine	Corticosteroids	Alive
Piñana E et al^21^	11‐year‐old boy	Sulfasalazine/naproxen	Corticosteroids	Alive
Lau G et al^22^	34‐year‐old woman	Sulfasalazine	Corticosteroids	Died
Daoulah A et al^23^	56‐year‐old woman	Sulfasalazine	Corticosteroids	Died
Ng CT et al^24^	17‐year‐old boy	Trimethoprim‐sulfamethoxazole	MARS	Alive
Yusuf IH et al^25^	15‐year‐old girl	Sulfasalazine	Corticosteroids	Alive
Girelli F et al^26^	53‐year‐old woman	Sulfasalazine/amoxicillin	Corticosteroids	Alive
Hernández N et al^27^	60‐year‐old woman	Sulfasalazine	Corticosteroids	Alive
Zaïem A et al^28^	45‐year‐old woman	Sulfasalazine	Corticosteroids	Alive
Ferrero NA et al^29^	15‐year‐old boy	Sulfasalazine	Corticosteroids	Alive
Pirklbauer M et al^30^	A 53‐year‐old woman	Sulfasalazine	Corticosteroids	Alive

The management of DRESS syndrome is challenging. It is important to withdraw the suspected drug, and the delay is associated with poorer outcomes.^31^, ^32^, ^33^ Supportive therapy in the intensive care unit should be provided to stabilize the patient. Early administration of systemic corticosteroid therapy is generally recommended.^34^ A systemic corticosteroid helps to improve both clinical symptoms and laboratory abnormalities within days.^34^ Most of the case reports of DRESS syndrome with liver dysfunction showed success with corticosteroid treatment (Table 1).

Liver transplantation is an option when the DRESS syndrome is associated with acute fulminant hepatic failure, but the prognosis remains poor (Table 2).^35^, ^36^, ^37^, ^38^, ^39^ Besnard et al^35^ reported two pediatric Crohn's disease patients undergoing liver transplantation after sulfasalazine‐induced DRESS syndrome. During follow‐up, one of them developed acute rejection and fatal aspergillosis. Amante et al^36^ and Roales‐Gómez et al^39^ reported adult patients treated with OLT, with no information on long‐term follow‐up. Mennickea et al^37^ reported an adult patient treated with OLT, with mortality in the postoperative period due to massive intra‐abdominal blood loss. Song et al^38^ reported living‐donor liver transplantation in a 14‐year‐old patient. The patient showed chronic rejection after a 25‐month follow‐up. A living donation would be an alternative to the OLT, mainly in case of the scarce availability of organs in timely fashion.

**Table 2:** Patient diagnosed with DRESS undergoing liver transplantation

Case report	Patient	Drug	Follow‐up
Besnard M et al^35^	10‐year‐old boy	Sulfasalazine	Died
Besnard M et al^35^	10‐year‐old girl	Sulfasalazine	Alive
Amante MF et al^36^	21‐year‐old woman	Lamotrigine	Unknown
Mennickea M et al^37^	60‐year‐old man	Sulfasalazine/vancomycin	Died
Song S et al^38^	14‐year‐old girl	Vancomycin	Alive
Roales‐Gómez V et al ^39^	22‐year‐old man	Ibuprofen	Alive
Present study	18‐year‐old boy	Sulfamethoxazole	Died

Recent studies support the use of Molecular Adsorbents Recirculation System (MARS), which uses albumin dialysis to mainly replace the liver's detoxification function as a rescue for liver failure patients. Roales‐Gómez et al^39^ described MARS use, although the patient did not respond well, and patients eventually underwent OLT. Ng et al^24^ reported a pediatric patient that underwent MARS in the intensive care unit with a satisfactory response.

This study showed a patient with sulfasalazine and trimethoprim‐sulfamethoxazole severe reaction. Sulfasalazine and trimethoprim‐sulfamethoxazole carry a significant risk of drug toxicity. Yusuf et al^25^ reported the first case of DRESS syndrome in a child treated for toxoplasma retinochoroiditis. Rare cases of immunoallergic reactions to sulfasalazine, including DRESS syndromes, have been reported, such as the classic “3‐week sulfasalazine syndrome” occurring three weeks after the first administration.^9^ The treatment of this reaction with a hefty dose of steroids, which can depress the immune system and can flare infections, most likely impacted the postoperative outcomes in the present case.

## 4 CONCLUSION

DRESS syndrome associated with acute liver failure is a life‐threatening condition. Liver transplantation is an option for the management of these patients, although the prognosis remains poor.

## CONFLICT OF INTEREST

The authors have no conflict of interest.

## AUTHORS CONTRIBUTION

Igor Lepski Calil: analyzed and interpreted the data. Rafael Soares Nunes Pinheiro: acquired the data and drafted the article. Ryan Yukimatsu Tanigawa and Francisco Tustumi: drafted the paper. Rafael Antônio Arruda Pecora: revised the paper critically for relevant intellectual content. Ruy Jorge Cruz Junior: revised the paper critically for valuable intellectual content. Luiz Augusto Carneiro D'Albuquerque: conceptualized and designed the study. Jorge Henrique Bento de Sousa: approved the final version to be submitted.

## ETHICAL APPROVAL

Local Ethics Committee approved the study.
